# 单孔与单操作孔胸腔镜下胸腺瘤切除术的临床疗效比较：一项倾向性评分匹配研究

**DOI:** 10.3779/j.issn.1009-3419.2022.101.15

**Published:** 2022-04-20

**Authors:** 兴国 杨, 磊 于, 振 余, 翔 高, 鑫 杜

**Affiliations:** 100730 北京，首都医科大学附属北京同仁医院胸外科 Department of Thoracic Surgery, Beijing Tongren Hospital, Capital Medical University, Beijing 100730, China

**Keywords:** 单孔, 胸腺瘤, 胸腔镜, 倾向性评分匹配, Single-port, Thymoma, Video-assisted thoracoscopic surgery, Propensity-score matching

## Abstract

**背景与目的:**

近年来随着临床微创技术的不断进步，胸腔镜胸腺瘤切除术经历了从三孔操作到单操作孔、单孔的发展历程。然而，单孔胸腔镜技术的可行性及安全性尚未得到普遍认可。本研究拟探讨单孔胸腔镜手术在胸腺瘤切除中的安全性与可行性，以期为临床手术方式的选择提供参考。

**方法:**

回顾性分析2018年1月-2021年9月在北京同仁医院接受胸腔镜胸腺瘤切除手术治疗的197例患者的临床资料。按照手术方式将患者分为单孔胸腔镜胸腺瘤切除组(单孔组，*n*=42)和单操作孔胸腔镜胸腺瘤切除组(单操作孔组，*n*=155)。经倾向性评分匹配，单操作孔组获得与单孔组术前基线资料具有可比性的42例患者，其中单孔组男性17例、女性25例，年龄28-72(48.00±9.43)岁; 单操作孔组男性20例、女性22例，年龄30-75(50.38±9.83)岁。对比两组的临床疗效。

**结果:**

两组均顺利完成手术，均无中转开胸或增加手术切口。单孔组和单操作孔组比较，胸腔引流时间和住院时间更短[(2.95±0.76) d *vs* (3.33±0.85) d; (4.57±0.83) d *vs* (5.07±1.13) d]，术后24 h及72 h视觉疼痛评分更低[(3.64±0.85)分*vs* (4.05±0.66)分; (2.33±0.75)分*vs* (3.07±0.68)分]，差异有统计学意义(*P* < 0.05)。单孔组、单操作孔组在手术时间[(130.00±26.23) min *vs* (135.24±27.03) min]、术中出血量[(69.52±22.73) mL *vs* (82.38±49.23) mL]等方面差异无统计学意义(*P* > 0.05)。

**结论:**

单孔胸腔镜胸腺瘤切除术是一种安全、可行、更微创的术式，术后疼痛较多孔胸腔镜手术减轻，恢复更快。

## Abstract (en)

**Background and objective:**

In recent years, with the advancement of minimally invasive techniques, thoracoscopic thymoma resection has experienced a development process from three-port video-assisted thoracic surgery (VATS) to two-port (TP) and single-port (SP) variants. However, the feasibility and safety of SP-VATS have not been generally recognized. This study intends to explore the safety and feasibility of SP-VATS in thymoma resection, in order to provide a reference for clinical surgicalselection.

**Methods:**

The clinical data of 197 patients who underwent thoracoscopic thymoma resection in Beijing Tongren Hospital from January 2018 to September 2021 were retrospectively analyzed. The patients were divided into SP-VATS group (*n*=42) and TP-VATS group (*n*=155). After matching propensity scores, there is no statistically significant difference in preoperative baseline data between SP-VATS group and TP-VATS group. Among them, there were 17 males and 25 females with an average age of 28-72 (48.00±9.43) years in the SP-VATS group, and 20 males and 22 females with an average age of 30-75 (50.38±9.83) years in TP-VATS group. The clinical effects of the two groups were compared.

**Results:**

The operation was successfully completed in both groups, and there was no conversion to thoracotomy or increased surgical incisions. Compared with the TP-VATS group, the chest drainage time and hospital stay in the SP-VATS group were shorter [(2.95±0.76) d *vs* (3.33±0.85) d; (4.57±0.83) d *vs* (5.07±1.13) d], and the visual pain score at 24 h and 72 h after surgery were lower [(3.64±0.85) points *vs* (4.05±0.66) points; (2.33±0.75) points *vs* (3.07±0.68) points] (*P* < 0.05). There was no statistically significant difference between the two groups in operation time [(130.00±26.23) min *vs* (135.24±27.03) min], intraoperative blood loss [(69.52±22.73) mL *vs* (82.38±49.23) mL] (*P* > 0.05).

**Conclusion:**

SP-VATS in thymoma is a safe, feasible, and less invasive procedure, with less postoperative pain and faster recovery than multi-port VATS.

手术切除是治疗胸腺瘤的主要手段^[1]^。传统开胸手术是最常见术式，然而其造成的创伤较大，患者术后康复较慢^[2]^。电视胸腔镜手术因其具有手术创伤小、术后疼痛轻、并发症较少、患者恢复快等优点，已经逐渐取代传统开胸手术，成为临床治疗胸腺肿瘤的首选术式^[3]^。三孔胸腔镜胸腺瘤切除术为电视胸腔镜手术中的常规经典术式，在治疗胸腺肿瘤中已取得较好疗效。但据临床研究^[4]^发现，部分接受三孔胸腔镜胸腺瘤切除术治疗的患者存在肋间神经受损、术后早期胸痛等问题，影响患者术后康复。近年来，随着临床微创技术的不断进步，胸腺瘤切除术经历了从三孔操作到单操作孔、单孔操作的发展历程^[5, 6]^。单孔胸腔镜胸腺瘤切除减少了切口数量，存在能够降低患者手术创伤、促进早日康复的理论可能^[7]^。但该技术的安全性、可行性及肿瘤学长期效果方面，还需要更多的研究去验证。本研究通过探讨单孔胸腔镜和单操作孔胸腔镜术式在胸腺瘤切除中的应用，以期为临床手术方式的选择提供参考。

## 1 资料与方法

### 1.1 一般资料

选择2018年1月-2021年9月在首都医科大学附属北京同仁医院胸外科同一诊疗组接受胸腔镜胸腺瘤切除患者的临床资料。纳入标准：①计算机断层扫描(computed tomography, CT)显示前纵隔占位，术后常规病理证实为胸腺瘤; ②手术初始方案为单孔胸腔镜方式或单操作孔胸腔镜方式。排除标准：①合并难控制的心肺功能不全，不能耐受全身麻醉单肺通气; ②合并重症肌无力的患者，术前评估吞咽、咀嚼与呼吸活动的肌力未满意控制，术后发生呼吸衰竭、肌无力危象风险大; ③既往有胸部手术史或进入胸腔后发现胸腔广泛致密黏连、需花费较长时间分离黏连。根据以上标准，最终共纳入197例患者，根据手术方式分为单孔胸腔镜胸腺瘤切除组(单孔组)和单操作孔胸腔镜胸腺瘤切除组(单操作孔组)。单孔组42例，其中男性17例，女性25例，年龄28-72(48.2±9.4)岁，其中经左侧入路11例，经右侧入路31例。单操作孔组155例，病例基线资料经倾向性评分匹配后获得与单孔组差异无统计学意义的42例患者，其中男性20例，女性22例，年龄30-75(50.0±10.2)岁，见表 1。

**表 1:** 倾向性评分匹配后两组患者临床资料对比 Comparison of clinical data between two groups after matching propensity score

Items	Single-portal group (*n*=42)	Two-portal group (*n*=42)	*t*/*χ*^2^	*P*
Gender			0.435	0.510
Male	17	20		
Female	25	22		
Age (Mean±SD, yr)	48.00±9.43	50.38±9.83	1.133	0.261
BMI≥25 kg/m^2^	14	18	0.808	0.369
MG	6	7	0.343	0.558
Laterality			0.233	0.629
Left	11	13		
Right	31	29		
Lesion size (Mean±SD, cm)	3.21±0.83	3.42±0.92	1.061	0.292
WHO classification			1.821	0.769
A	4	6		
AB	11	9		
B1	7	9		
B2	16	12		
B3	4	6		
Masaoka stage			2.041	0.841
I	17	14		
IIa	13	10		
IIb	5	7		
IIIa	5	8		
IIIb	1	2		
IVa	1	1		
BMI: body mass index; WHO: World Health Organization; MG: myasthenia gravis.				

### 1.2 手术方法

所有患者入手术室，由麻醉医生完成双腔气管插管全身麻醉。仰卧位，根据患者肿瘤偏向胸骨中线的位置选择左侧或右侧入路，患侧(肿瘤偏向侧)抬高45°。单孔组于腋中线偏前第4肋间作2 cm-4 cm切口，应用一次性切口保护套保护切口，使用德国STORZ电视胸腔镜系统，5 mm 30°胸腔镜(图 1)。单操作孔组于腋中线第4肋间作1 cm切口为观察孔，使用10 mm 30°胸腔镜，胸腔镜引导下于腋前线第4肋间作2 cm-3 cm切口为操作孔。两组患者均行全胸腺切除及胸腺瘤切除：保护膈神经，于其前方心包反折处打开纵隔胸膜，从胸腺下极开始，超声刀及电勾钝锐性向上游离直至无名静脉水平; 自胸骨后打开纵隔胸膜，钝锐性将胸腺从胸骨面完全游离; 于无名静脉前仔细解剖，找到胸腺滋养血管，超声刀切断，充分游离胸腺上极; 将胸腺及胸腺瘤完整切除; 用标本取物袋将所切组织取出。对于合并重症肌无力患者清扫前纵隔各组脂肪。如肿瘤侵犯心包、肺、膈神经、无名静脉等，则将受侵组织切除。如术中探查胸腔内肿瘤种植转移，则将转移灶全部切除。术后于观察孔放置24 F胸腔闭式引流管1根。

**图 1:**
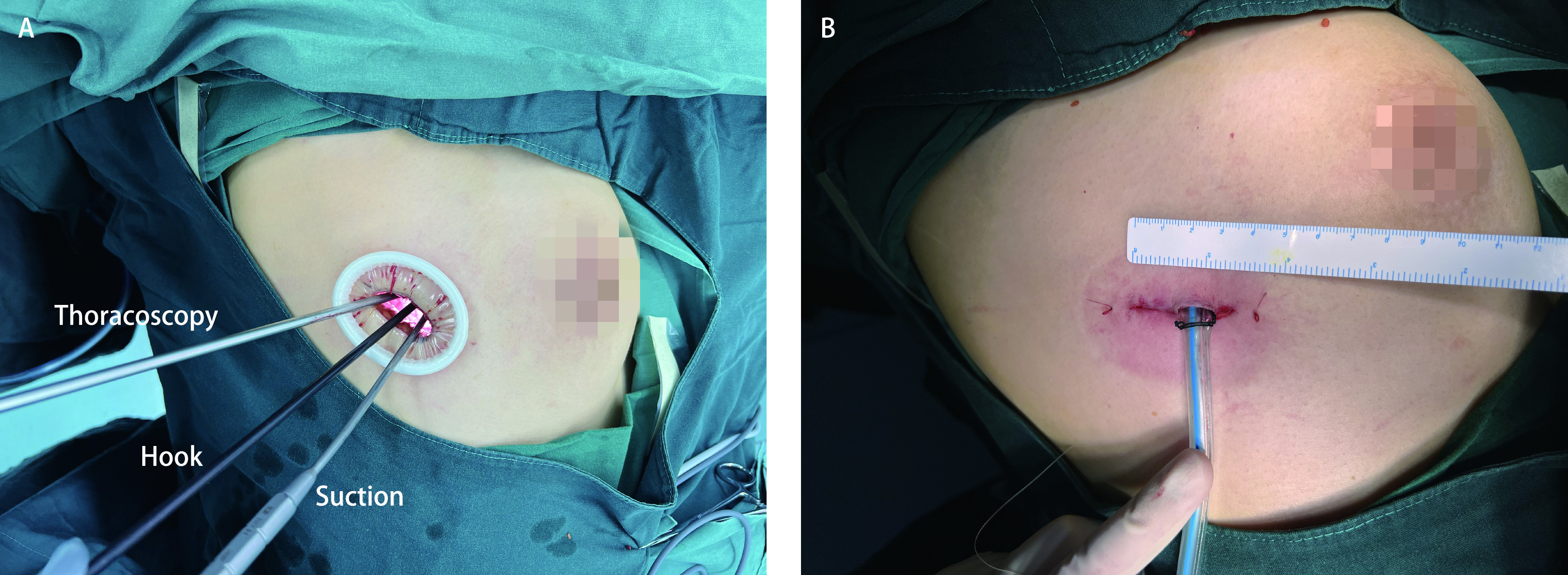
手术切口。A：切口内放置胸腔镜镜头、电钩和吸引器; B：切口位于腋中线偏前第4肋间，长2 cm-4 cm。 Surgical incision. A: The thoracoscopic lens, hook and suction are placed into the incision; B: The surgical incision is a 2 cm-4 cm incision in the 4^th^ intercostal space anterior to midaxillary line.

### 1.3 术后处理与观察

对于合并重症肌无力的患者术后常规入重症监护室观察，术后应用溴比斯的明控制症状，密切关注病情变化，注意早期发现和鉴别肌无力危象。所有患者术后24 h及72 h测定视觉模拟疼痛评分(visual analogue scale, VAS)，评价患者术后疼痛感，得分范围0分-10分，得分越高，表明患者疼痛感越强烈。术后拔除胸腔引流管指征：咳嗽时无漏气，胸腔24 h引流量小于100 mL，复查胸片提示肺复张良好，胸腔无积液。

观察并记录各组患者手术时间、术中出血量、引流管留置时间、术后24 h及72 h VAS评分、术后并发症和术后住院时间等指标。

### 1.4 统计学方法

使用SPSS 25.0进行统计分析，计量资料采用均数±标准差表示，组间比较采用*t*检验。计数资料采用例数表示，组间比较采用*χ*^2^检验及*Fisher*确切概率法。以*P* < 0.05为差异具有统计学意义。

## 2 结果

两组患者年龄、性别、体质指数差异均无统计学意义(*P* > 0.05)，见表 1。两组之间的肿瘤的位置、病理类型、肿瘤大小、临床分期均差异无统计学意义(*P* > 0.05)。具体数据见表 1。术前合并重症肌无力患者13例：其中单孔组6例，单操作孔组7例; Osserman IIB型3例，Osserman IIA型1例，眼肌型肌无力I型7例。所有Osserman IIB型患者在术前1周按照0.4 g/kg输注丙种球蛋白5 d，改善呼吸、咳嗽与吞咽症状后手术，Osserman I型患者则口服溴吡斯的明治疗。术后全组均未出现肌无力危象。

84例患者均顺利完成手术，无围手术期死亡，无中转开胸或增加手术切口。单孔组术后引流管留置时间及术后住天数短于单操作孔组，术后24 h及72 h VAS评分均明显低于单操作孔组，差异有统计学意义(*P* < 0.05)。单孔组的手术时间、术中出血量等指标差异无统计学意义(*P* > 0.05)。见表 2。

**表 2:** 两组术中及术后资料对比 Comparison of intraoperative and postoperative data between two groups

Items	Single-portal group (*n*=42)	Two-portal group (*n*=42)	*t*/*χ*^2^	*P*
Length of surgery (Mean±SD, min)	130.00±26.23	135.24±27.03	0.901	0.370
Bleeding (Mean±SD, mL)	69.52±22.73	82.38±49.23	1.537	0.128
Thoracic drainage duration (Mean±SD, d)	2.95±0.76	3.33±0.85	2.167	0.033
Postoperative hospital stay (Mean±SD, d)	4.57±0.83	5.07±1.13	2.304	0.024
Postoperative VAS pain score (Mean±SD)				
Postoperative 24 h	3.64±0.85	4.05±0.66	2.436	0.017
Postoperative 72 h	2.33±0.75	3.07±0.68	4.719	< 0.001
Complications			1.120	0.480
Arrhythmia	1	2		
Phrenic nerve injury	0	2		
Chest infection	2	1		
VTE	0	1		
VTE: venous thromboembolism; VAS: visual analogue scale.				

术后两组并发症见表 2。单操作孔组有2例发生膈神经损伤，因为术中发现肿瘤侵犯膈神经。术后单孔组1例、单操作孔组2例出现心房颤动或室上性心动过速等心律失常，降心率、吸氧等对症治疗后恢复正常。单孔组2例和单操作孔组1例术后胸管拔除后出现发热，查胸片提示胸腔少量积液，给予抗炎对症治疗后，患者体温自行恢复正常。术后1例发现下肢肌间静脉血栓，给予口服利伐沙班抗凝治疗，出院2周后复查下肢静脉超声，下肢静脉血栓消失。单孔组发生并发症患者比例少于单操作孔组，但差异无统计学意义(*P* > 0.05)。

## 3 讨论

胸腺瘤是最常见的原发性前纵隔肿瘤，占成人所有纵隔肿瘤的30%，发病率为1.5/100万人年^[8]^。手术治疗是胸腺瘤治疗最为重要的治疗方式，手术应遵循外科学解剖切除和肿瘤学根治性切除的原则，推荐行全胸腺切除，合并重症肌无力的患者应该行扩大的胸腺切除术^[9]^。相比于传统开胸，胸腔镜胸腺瘤切除手术治疗Masaoka-Koga Ⅰ期胸腺瘤具有相同的远期肿瘤学效果，然而手术时间更短，术中出血量更少，术后恢复更快，近年来成为早期胸腺瘤切除的主要手术方式^[10]^。单孔胸腔镜胸腺瘤切除进一步减少了手术切口。有研究^[11]^认为，与多孔胸腔镜手术相比，单孔胸腔镜胸腺瘤切除手术时间更短，术后引流量更少，术后带管时间及住院时间更短，术后疼痛更轻微。

单孔胸腔镜手术只有1个切口，合适的切口选择有利于手术操作及术中暴露，降低手术难度。我们的切口选择左右侧均在第4肋间腋中线略偏前的位置，相比单操作孔组在手术上具有以下优势：①此处的切口较单操作孔镜孔的位置偏前，有利于观察无名静脉上方的结构，如胸腺上极、甲状腺下静脉及变异血管等; ②切口位置的选择和单孔肺叶切除切口位置相近，靠近肺门，在同时合并做肺楔形、肺叶切除时具有明显优势; ③部分胸腺瘤病例合并胸腔内肿瘤播散，而播散位置多在肋膈角、脊柱旁沟、膈肌附近，单孔胸腔镜相比单操作孔位置靠后，有利于观察及切除此处的转移瘤。单孔胸腔镜手术较单操作孔手术对术者的要求更高，需要有多孔胸腔镜胸腺瘤手术的经验。手术者及扶镜手均站在患者的背侧，为避免扶镜手对术者造成干扰，我们的经验是扶镜手站在头侧，胸腔镜身放在切口的最后方，双方操作都能不受影响。然而，单孔胸腔镜位置靠后，在处理胸腺上极时手术器械距离更远，难度更大。我们的经验是助手使用较细的有弧度的牵拉钳牵拉胸腺下极，这样使胸腺的上极向下暴露，术者使用细头弯吸引器钝性游离胸腺被膜及周边组织，既安全又方便。

手术中应严格注意无瘤原则，因为胸腺瘤易通过胸腔播散，对于一些恶性程度高的胸腺瘤，可能发现肿瘤有破溃，应积极行胸腔探查，寻找胸腔转移灶，并将转移灶全部切除，同时做胸腔热灌注治疗^[12]^。有研究^[13, 14]^认为，为了最大限度地降低肿瘤播散的风险，单孔胸腔镜胸腺切除手术应选择包膜完整直径 < 4 cm胸腺瘤，对于肿瘤 > 4 cm和无名静脉受侵犯被认为是单孔胸腔镜的禁忌证。Odaka等^[15]^的研究显示对于直径≥50 mm的胸腺瘤而言，胸腔镜手术能够达到和开胸手术相似的肿瘤学效果，胸腺瘤大小不是胸腔镜禁忌证，能否使用胸腔镜更取决于胸腺瘤的Masaoka分期及肿瘤与周围血管的关系。本研究中，两组患者均完整切除胸腺瘤及胸腺组织，单孔组切除了3例直径超过5 cm的胸腺瘤，所有手术均安全顺利完成。我们的体会是单孔胸腺瘤切除可以达到和单操作孔一样的肿瘤学效果，肿瘤的大小并非是单孔手术的绝对禁忌。

如术中发现胸腺瘤未侵犯对侧纵隔胸膜，则应避免其发生破损，以防止肿瘤向对侧胸腔播散。笔者曾于门诊接诊多例胸腔镜或正中胸骨劈开胸腺瘤切除患者，术后复查发现肿瘤双侧胸膜种植转移。近年来，相比于单侧肋间入路胸腔镜手术，越来越多的胸外科医生采用剑突下手术入路^[16, 17]^。有研究^[18]^报道，剑突下入路胸腺切除具有术后疼痛轻、伤口美观、对对侧纵隔脂肪和膈神经暴露解剖清晰的优势。然而，剑突下入路需打开双侧纵隔胸膜，如术中发生胸腺肿瘤破溃，形成双侧胸腔种植，将会造成非常严重的后果。另外，双侧胸腔的操作会使胸膜发生黏连，为将来再次进胸手术增加困难。剑突下入路长期肿瘤学效果和患者远期能否受益尚需更多的研究随访来验证。

本研究结果发现，单孔组术后疼痛评分明显小于单操作孔组，差异有统计学意义(*P* < 0.05)。分析原因如下：首先，单操作孔组比单孔组要多出腋中线的切口，手术影响了两个位置，对患者组织、肌肉、血管等损伤加重，加重了疼痛; 其次，腋中线切口相对腋前线肋间隙更窄，单操作孔操作时胸腔镜身对腋中线切口肋间神经卡压更明显。术后单操作孔组胸管需放在此处，也在一定程度上加重了疼痛。研究表明单孔胸腔镜较两孔、多孔胸腔镜手术后患者疼痛减轻，明显提高其术后生活质量。随着人们生活水平的逐步提高，对伤口的美观有更高的要求，单孔组术后胸壁仅有一个长约3 cm的切口，位于相对隐蔽的腋下。而单操作孔组的操作口位于腋前线，靠近乳头，术后形成疤痕位置显眼不美观。如患者是女性，此切口会通过乳腺，术后发生伤口感染的概率也增高。Hicham等^[19]^对351例患者行单孔胸腔镜手术治疗，其中85%的患者对单孔术后疤痕表示满意。

本研究单孔组的术后引流管留置时间及术后住院天数均短于单操作孔组，差异有统计学意义(*P* < 0.05)。这与既往研究^[11, 20]^得出的结果相同。分析原因，单孔组减少了腋中线手术切口，减少了术后伤口渗血渗液量，或许有助于更早地拔除胸腔引流管。单孔组术后住院时间更短，恢复更快，有助于降低患者医疗费用。

综上所述，单孔胸腔镜胸腺瘤切除手术是一种安全可行的技术，与单操作孔胸腔镜胸腺瘤切除手术相比，具有术后疼痛小、恢复更快的优点。值得注意的是，本研究仅涉及围手术期短期指标，对术后患者长期生存率、长期生活质量等方面未涉及。这些都有待于今后进一步的研究。
